# Social Determinants as Mediators of the Emotional State of People With Type 2 Diabetes and/or Hypertension During the COVID‐19 Pandemic in Ecuador and Spain

**DOI:** 10.1111/hex.70123

**Published:** 2024-12-11

**Authors:** María José Sanchís‐Ramón, Elisa Chilet‐Rosell, Andrés Peralta, Marta Puig‐García, María Fernanda Rivadeneira, Cintia Caicedo, Ikram Benazizi‐Dahbi, Blanca Lumbreras, Montse Nicols, Ana Cebrián, Wifredo Ricart, Ester Lopez‐Miras, Lucy A. Parker

**Affiliations:** ^1^ Departamento de Salud Pública Universidad Miguel Hernández de Elche Sant Joan d'Alacant Spain; ^2^ Centro de Investigación Biomédica en Red Epidemiología y Salud Pública (CIBERESP) Madrid Spain; ^3^ Instituto de Salud Pública Pontificia Universidad Católica del Ecuador Quito Ecuador; ^4^ Centro de Epidemiologia Comunitaria y Medicina Tropical (CECOMET) Esmeraldas Ecuador; ^5^ Centro de Salud de Alzira Valencia Spain; ^6^ Centro de Salud Cartagena Casco Murcia Spain; ^7^ Fundació Institut d'Investigaciò Biomèdica de Girona‐ IDIBGI Girona Spain; ^8^ Centro de Investigación Biomédica en Red Obesidad y Nutrición (CIBEROBN) Madrid Spain; ^9^ Unitat de Diabetis, Endocrinologia i Nutrició (UDENTG) Departament de Salut Generalitat de Catalunya

**Keywords:** COVID‐19 pandemic, diabetes mellitus, emotional state, social determinants of health, social policies

## Abstract

**Introduction:**

We aimed to explore the impact of the COVID‐19 pandemic and the resulting restrictions on the emotional state of people with type 2 diabetes mellitus (T2DM) and/or hypertension in Ecuador and Spain. Given the differences in sociopolitical and socioeconomic contexts between these two countries, the research focused on how these diverse environments and their management of social policies and pandemic strategies influenced the emotional well‐being of individuals with chronic illnesses.

**Methods:**

We conducted 36 semi‐structured telephone interviews between August and December 2020 with adults diagnosed with T2DM and/or hypertension (19 in Ecuador, 17 in Spain). The interviews were recorded, anonymized and transcribed for thematic analysis. This approach allowed us to systematically identify and analyse themes related to the participants' emotional experiences during the pandemic.

**Results:**

The results revealed a significant deterioration in the emotional state of participants, attributable to the stress generated by the health crisis and concerns related to their chronic illnesses. The situation elicited a range of emotions among participants, from boredom and apathy to fear, uncertainty and depression. The study highlighted how the impact on emotional well‐being was shaped by the interplay between conjunctural determinants (measures to control COVID‐19 infections) and structural factors driving inequalities (social class, gender, ethnicity).

**Conclusion:**

We developed a conceptual framework illustrating how measures to control COVID‐19 infections directly influenced economic, health and social determinants, which interacted with pre‐existing inequalities and had a differential impact on individuals' emotional well‐being. This framework can be useful for designing more effective and equitable social policies during future health crises, ensuring they address social needs and safeguard psychological and emotional well‐being, particularly among vulnerable groups such as those with chronic illnesses.

**Patient and Public Contribution:**

Thirty‐six participants diagnosed with T2DM and/or hypertension (19 in Ecuador, 17 in Spain) contributed to the study by sharing their emotional experiences during the pandemic. Their detailed accounts enriched the research by providing valuable insights into how the pandemic affected their emotional well‐being. There was no additional involvement or contribution from the public in the design, conduct, analysis or interpretation of the study, nor in the preparation of the manuscript.

## 1 Introduction

The preventive measures implemented during the COVID‐19 pandemic, such as mobility restrictions, lockdowns and social distancing, had significant financial, social and health consequences, and severely impacted global economic development [1]. As economic activity and employment plummeted, many individuals fell into poverty, with those already vulnerable before the pandemic being hardest hit [2].

The ‘refamiliarization’ of caregiving during the pandemic deepened the unequal distribution of gender roles. This led to a higher likelihood of poor general mental health and impaired well‐being among women, who were also more likely to lose their jobs or suffer income loss due to the pandemic [3, 4, 5]. Moreover, COVID‐19 restrictions resulted in reduced health coverage and weakened health services worldwide [6, 7]. People with non‐communicable diseases had limited access to routine care. In countries with weak social protection or welfare systems, shortages of material and human resources in the health sector had the greatest impact on vulnerable populations [8, 9].

Confinement measures led to social isolation, adversely affecting the emotional state of the general population [10, 11, 12, 13, 14, 15]. Individuals who faced difficulties accessing basic necessities like healthy food, healthcare and medication were more likely to experience psychosocial problems [16, 17]. Among people with diabetes, psychological issues such as anxiety and depression were reported more frequently than in the general population, affecting their healthcare needs [12, 13]. Psychological stress and a decreased sense of control over their chronic health conditions were linked to factors such as loneliness, lack of social and institutional support, limited information about COVID‐19 and restricted access to medical care [12, 18, 19].

Given these circumstances, it is relevant to explore the emotional state of individuals with T2DM and/or hypertension who were in various vulnerable situations during the COVID‐19 pandemic in countries with differing public and social security systems. T2DM and hypertension were chosen due to their chronic nature, requiring continuous medical monitoring and their frequent co‐occurrence. This study aims to explore the impact of the COVID‐19 pandemic and the resulting restrictions on the emotional state of individuals with type 2 diabetes mellitus (T2DM) and/or hypertension in Ecuador and Spain.

## 2 Material and Methods

### 2.1 Study Design

This study employed an exploratory qualitative design aimed at understanding the emotional impact of the COVID‐19 pandemic on individuals with type 2 diabetes mellitus (T2DM) and/or hypertension. We aimed to gain in‐depth insights into participants' experiences, rather than statistical generalisation. We conducted 36 semi‐structured telephone interviews between August and December 2020 with adults (18 men, 18 women) diagnosed with hypertension and/or T2DM in Ecuador (19 participants) and Spain (17 participants). Our study was part of the CEAD project, which aims to evaluate the implementation of comprehensive diabetes care in low‐resource settings.

To achieve a more comprehensive analysis of the situation and explore factors associated with the sociopolitical context, we expanded the scope of this analysis to include interviews of people living in Spain. Our intention was not to compare the two countries, but rather to consider the interaction of different contextual factors such as the welfare state. Ecuador was chosen as the primary site due to its relevance within the CEAD project, which focuses on diabetes prevention in low‐resource settings. Spain was included as the base country of the research team leading the project. The contrasting socioeconomic and sociopolitical contexts of both countries also provided valuable diversity for our analysis.

### 2.2 Conjunctural Context

The management of the COVID‐19 pandemic in Ecuador involved strict measures, including mandatory lockdowns and social distancing, enforced by police and military forces [14]. A state of emergency was declared on March 17th [20], due to the rapid surge in cases and the collapse of the public health system. The lockdown included long curfews and mobility restrictions, forced people to stay home except for essential needs like food or medicine. This situation led to unmet basic needs and exacerbated economic hardship, resulting in significant job losses, particularly in non‐essential sectors and informal work [21, 22].

In Spain, the measures to confront the pandemic were among the strictest in Europe. The Spanish government declared a state of alarm on March 14th [23], enforcing a national lockdown that restricted mobility, closed non‐essential businesses and limited outdoor activities. These measures significantly impacted the economy and emotional well‐being, leading to job losses in sectors like hospitality, tourism and retail, and severely affecting many Spanish families [23].

Ecuador's social protection system is based on principles of universality, equality and solidarity but is constrained by the economy's structural limitations and its low capacity to absorb labour and generate income [24]. Frequent changes in authorities further weaken institutional structures and hinder effective policymaking [25]. During the pandemic, the Public Health Ministry and the Ecuadorian Institute of Social Security struggled to design effective policies due to overwhelmed capacities, such as shortages of medicines and hospital beds [26].

Spain's welfare state features a universal health system, pension and unemployment protections, and minimum wage programmes for poverty prevention. However, it increasingly resembles other European welfare states that prioritise economic benefits over health services, elderly care and education [27]. The pandemic exposed and worsened the fragility of the Spanish public healthcare system, which had been weakened by years of austerity and budget cuts.

In both countries, the prevalence of chronic conditions such as type 2 diabetes and hypertension were notably high during the pandemic. According to the International Diabetes Federation's Atlas (2020) the prevalence of diabetes in Ecuador stood at 7.4% among adults, while in Spain, it was 10.3% [28]. Hypertension, a key comorbidity, affected 26.7% of adults in Ecuador and 19.8% [29] in Spain [30]. These rates highlight the significant health burden in both countries, making people with these conditions especially vulnerable to the adverse effects of the pandemic.

### 2.3 Sample Design and Data Collection Procedure

Participants were recruited through purposive sampling to ensure a heterogeneous group in terms of sex, age and socioeconomic status. We aimed for a balanced representation of both genders, selecting 18 men and 18 women to explore potential differences in emotional responses related to gender. Between 17 August and 31 December 2020, we conducted semi‐structured telephone interviews with individuals diagnosed with T2DM and/or hypertension, as face‐to‐face interviews were not possible due to mobility restrictions. The number of participants was gradually increased until data saturation was reached, at which point new interviews no longer provided significant information.

In Ecuador, we recruited people in a low‐income district in the south of Quito using patient support groups for people with T2DM in different primary care centers (*Chimbacalle*, *La Magdalena*, *Centro Histórico* and *Comité del Pueblo*). We also recruited in Esmeraldas, a rural region in the north where various ethnicities coexist in hard‐to‐reach communities. In Spain, we recruited people through different contacts in two health centers (Cartagena and Alzira) and one hospital (Girona) thus enhancing clinical heterogeneity by including both those seeking primary care and individuals with more complex pathologies.

The duration of the interviews ranged from 16 to 53 min and were conducted according to the participants' preferred times, considering the language difficulties of some participants (immigrants who do not speak Spanish or people from Esmeraldas who speak different languages). All interviews were conducted by individuals with health‐related degrees. In Ecuador, a team of four interviewers (one man and three women) received training in interview techniques. In Spain, healthcare professionals in the health facilities invited people to participate in the study and requested their oral consent to receive a phone call from one of three female researchers from Miguel Hernández University.

### 2.4 Data Collection Instrument

We used semi‐structured interviews using interview scripts comprised the following thematic blocks (see Supporting Information S1: Interview guide).

1. Sociodemographic characteristics of the interviewee.
2. Experience of the disease before the pandemic.
3. Management of the disease during the pandemic.
4. Personal experiences during COVID‐19 lockdown.
5. Possible measures for improving health and well‐being in health crisis situations.

The interview questions were developed based on existing literature related to chronic illnesses and their management. Given that the interviews were conducted during the early phase of the COVID‐19 pandemic, when there was limited research available, the questions were designed to explore various aspects of participants' experiences and perspectives. To establish reliability, interviews were conducted using a standardised protocol to ensure consistency in question administration. The same set of questions was utilised across all interviews, and recordings were transcribed verbatim to maintain accuracy in data interpretation.

### 2.5 Data Analysis

The interviews were recorded, transcribed, anonymized and analzed using Atlas.ti 8. Grounded theory methodology was employed to generate theories from the qualitative data [31], focusing on participants' subjective experiences in relation to social and health contexts. An open coding process identified emerging concepts related to emotions, challenges and determinants during the pandemic. As Aspers and Corte note, qualitative research emphasises the importance of interpretation in understanding individual experiences, which guided our analysis [32]. After several revisions, recoding and adjustments of codes, three researchers (M.J.S.R., M.P.G. and I.B.D.) carried out the triangulation phase by applying the list of codes. The team incorporated researchers from different disciplines (sociology and health sciences) and with varying levels of familiarity with the environments studied, thereby enhancing the credibility of the analysis [33]. Two of the researchers involved in the triangulation were also involved in the data collection. This phase included comparing codes among researchers and discussing discrepancies. Selective and axial coding to refine and organise the thematic codes into a theoretical structure [31, 34], aiming for an in‐depth understanding of how individuals with T2DM and/or hypertension experienced the pandemic emotionally and how social and health determinants influenced their experiences.

## 3 Results

Nineteen valid interviews were conducted in Ecuador and 17 in Spain, bringing the total to 36. The mean age was 61 years (range 43–84), and half of the participants were women (9 in Ecuador and 9 in Spain). The mean age of the female participants was 60 years (54 in Ecuador, 66 in Spain) and for the male participants it was 63 years (60 in Ecuador, 66 in Spain).

Thirty‐three participants were diagnosed with T2DM (17 in Ecuador, 16 in Spain), 23 with hypertension (10 in Ecuador, 13 in Spain) and 21 with both conditions (9 in Ecuador, 12 in Spain). The mean household size was three (5 in Ecuador, 2 in Spain); 53% of interviewees in Ecuador said they lived with five or more people in the same home, while 70% of interviewees in Spain said they lived alone or with their partner.

Five (26%) participants in Ecuador and 13(76%) in Spain reported a monthly household income above the basic monthly salary (BMS) (in 2020 this was 400 USD in Ecuador, 950 Euros in Spain). Three individuals in Ecuador reported having no income, while in Spain one participant in Alzira reported having no income and no schooling. Occupations varied, with many informal jobs in Ecuador, including street vendors, small‐scale farmers, cleaners, taxi drivers, doctors, mechanics, domestic assistants, clerks and supermarket cashiers. Two participants were unemployed (see Supporting Information S2 Table 1: Sociodemographic characteristics of the participants).

The key themes (and subthemes) extracted from the participants' discourses are organised to address the following broad questions:

1. What was the emotional state of people with T2DM and/or hypertension during the pandemic and what factors influenced their emotional state?
2. What areas of these people's lives were affected and in what way?

### 3.1 Emotional State of People With T2DM and/or Hypertension During the Pandemic and Associated Factors

Broadly speaking, we identified two main factors that affected the emotional state of people with T2DM and/or hypertension during the pandemic: (1) Their chronic condition and (2) the situation caused by the pandemic. These factors are closely related and mutually influential, but participants discussed them separately.

#### 3.1.1 Impact of the Chronic Condition

Interviewees' health status (T2DM and/or hypertension) caused divergent emotions, ranging from tranquility, carefreeness and/or optimism (‘*The diseases hardly bother me*’ [ID11] Man, age 57, T2DM and hypertension, Girona, Spain) to the opposite extreme of depression, emotional exhaustion, worry and/or helplessness. Men and women alike (seven men, seven women) indicated that their chronic illness negatively affected their emotional state:

…it's a…very sad disease I wouldn't…I wouldn't wish it on anyone.

[ID36] Man, age 59, T2DM, Quito, Ecuador

##### 3.1.1.1 Impact of the Pandemic

The pandemic significantly affected the emotional state of almost all interviewees, who expressed emotions ranging from apathy and boredom, to depression, nervousness, fear and worry, as well as nostalgia for moments and situations experienced before the pandemic (e.g., socialising and freedom of movement) that they felt they had lost without knowing when or how, or if they would get them back:

Uff! Sometimes I think ‘I wouldn't mind dying, I'm already buried alive’ What a life!…that's what I think.

[ID13] Woman, age 73, T2DM and hypertension, Spain

…it's barely affected my physical health, but emotionally yes, as I said before, it has affected me a lot.

[ID22] Woman, age 43, T2DM, > BMS, Esmeraldas, Ecuador

#### 3.1.2 The Combination of Both Scenarios: Chronic Disease and Pandemic

The combination of both scenarios made the interviewees aware of the risk they were facing. They saw the pandemic as a warlike scenario where they were more vulnerable than the rest of the population and had to make a greater effort to protect themselves from the enemy (COVID‐19). People in their immediate environment (family, health professionals, work colleagues) intensified this feeling of vulnerability, reminding them of their position as an at‐risk person:

…I'm on the front line in this pandemic and I have to stay in the trenches and not stick out because otherwise I am exposed to picking it up and leaving forever.

[ID06] Man, age 80, T2DM and hypertension, > BMS, Spain

…there is no way out of here either, it's scary because they say diabetics catch this disease quickly, so we have to look after ourselves.

[ID21] Woman, age 63, T2DM and hypertension, < BMS, Quito, Ecuador

Being constantly reminded that they are at risk during the pandemic translated into fear of contagion. Although people from both countries experienced this fear, we observed trends associated with the country of residence and the sex of the interviewee. Fear for oneself appeared to be more frequently reported in Ecuador, perhaps due to the perceived shortage of economic, health and food resources which caused them to internalise their physical vulnerability to a greater extent and be more aware of the consequences that COVID‐19 could have on them:

…here we lacked so much economically, both diabetes and blood pressure medicine.

[E21] Woman, age 63, T2DM and hypertension, <BMS, Quito, Ecuador

In Spain, the men expressed greater concern about the possible financial and health consequences for their family or loved ones, while women were more concerned about not being there to care for the family or being a source of contagion:

…if I were to be absent [to die], I know there would be a problem. A big problem because half of the income I have now would cease to exist….

[E02] Man, age 71, T2DM and hypertension, > BMS, Spain

…I am a widow […] if anything happens to me, they'll be left with nobody. […] Well, I work in a shopping center. So, when I come home, the first thing I do is take off my clothes, my shoes, leave them outside and everything, because I don't want to give it to my children.

[ID17] Woman, age 52, T2DM, > BMS, Spain

### 3.2 Areas of Vulnerability in People With T2DM and/or Hypertension During the Pandemic

#### 3.2.1 Economic Sphere: Employment and Family Finances

Participants had different perceptions of how the health crisis had affected their financial situation. In Spain, with few exceptions, participants considered that their financial situation had not changed, or had even improved because they had fewer expenses during lockdown. Of note, most interviewees in Spain received a retirement pension:

…the truth is [the family finances]. haven't been affected. Also, because we spend less.

[ID03] Man, age 62, T2DM and hypertension, > BMS, Spain

However, in Ecuador, the interviewees indicated that their financial situation had worsened because their monthly salary had been reduced or delayed (among people in formal work) or because they were unable to maintain their jobs and/or means of subsistence, due to mobility restrictions, so had no income.

…right now there is no work, the only work is going out and selling on the street, and we can't do that.

[ID28] Woman, age 55, street vendor, no income, Quito, Ecuador

Added to this were the costs of medications and in some cases private medical consultations. Most participants said they had received no financial or social aid and several people said they were surviving with the help of family members. One participant illustrated the struggle to secure basic needs due to lack of income:

I mean, sometimes we're… mmm, doing forced intermittent fasting. Yes, sometimes we don't have enough for a single day, I mean, we eat […] just once.

[ID26] Man, age 65, six cohabitants, < BMS, Quito, Ecuador

### 3.3 Health Sphere: Access to Health Services and Medication Partly Modulated by People's Financial Situation

During the most critical phase of the pandemic, people in both countries had difficulty accessing healthcare; however, several interviewees in Ecuador perceived a deterioration in the functioning of the Ecuadorian healthcare system. They described a lack of routine care for their chronic disease and shortages of medication and staff. Some interviewees said that despite their poor financial status, they had to pay for private healthcare:

…health is only for those who have money, and we have no choice but to use what we don't have to survive.

[ID25] Woman, age 48, T2DM, Quito, Ecuador

In Spain, prescriptions were signed and processed electronically, and the medications were free. In contrast, a few interviewees in Ecuador said they obtained the medication for free, but most said they had to pay a high price, and that the medication was difficult to locate owing to shortages in pharmacies, health centers and hospitals.

…well right now since there are no medications anywhere, I'm buying pills….

[ID21] Woman, age 63, T2DM and hypertension, < BMS, Quito, Ecuador

Limitations and barriers to accessing healthcare, routine check‐ups and medication for chronic diseases made individuals feel they had to take full charge of their health:

…with the daily blood tests that I do, well, my sugar spikes a little and then I have to restrict myself for two or 3 days and go almost hungry […] for my glucose to go down.

[ID02] Man, age 71, T2DM and hypertension, Spain

In the rural setting of Ecuador, this responsibility manifested as anxiety, nervousness, uncertainty and helplessness because resources to monitor blood glucose were scarce and because people had limited access to healthy food that would help control their disease.

… yes, I was quite scared, my nerves were on edge, and after my cousin died, who had the same illness […] and died of COVID, then after that, I was afraid of going through the same thing….

[ID22] Woman, age 43, T2DM, > BMS, Esmeraldas, Ecuador

… until the last appointment, they gave me the [medication] I take, but from there […] I don't know what to do anymore because they told me they couldn't attend to me […] that's where I have this uncertainty, I don't know what's going to happen.

[ID 23] Man, age 53, T2DM, Quito, Ecuador

#### 3.3.1 Social Sphere: Social Relationships (Family, Friends) and Support Networks

One of the most frequently mentioned issues was the mobility restrictions. Participants said that their financial situation as well as their physical and emotional well‐being were closely related to these restrictions and the impossibility of socialising with other people:

Yes, because before I went out more and walked. But since this, well I even feel more tired, I don't know if it also has a more emotional or psychological component….

[ID06] Man, age 80, T2DM and hypertension > BMS, Spain

Bored, of having to be stuck in here, and more eager to go out than before.

[ID14] Woman, age 67, T2DM and hypertension, > BMS, Spain

…imagine three, 2 months stuck inside, 3 months without working, without being able to do anything. So, it's…pretty serious, it's terrible!.

[ID36] Man, age 59, T2DM, Quito, Ecuador

Mobility restrictions also affected family relationships, and this clearly had an impact on the emotional state of the interviewees, causing episodes of sadness and longing and even anger and helplessness:

Well, I think the same as all families. Not being able to hug your child, to kiss them, to be close to them.

[ID09] Woman, age 57, T2DM and hypertension, > BMS, Spain

…the helplessness, the anger you feel because you can't even give them a hug, a word of encouragement, it's really messed up for a person who is attached, close to their family, isn't it?

[ID20] Man, age 50, T2DM, < BMS, Quito, Ecuador

However, the effects were not all negative: some interviewees said they noticed during the pandemic that their family cared for them more, which strengthened their emotional state because it showed they mattered to other people:

…what I've noticed is that they have looked after me more.

[ID04] Woman, age 75, T2DM and hypertension, < BMS, Spain

In addition, some interviewees explained that although it was lockdown that forced the family together, the result was improved communication in the family unit, in some cases from a situation of almost no communication:

…it seems to me that this situation has united us as sisters, as a family.

[ID22] Woman, age 43, T2DM, > BMS, Esmeraldas, Ecuador

Some interviewees in Spain also said that social relations (with friends) had weakened or broken down completely, causing sadness and nostalgia:

Now, social relations, in the strict sense of friendships etc., have been affected, absolutely. I have zero social life.

[ID16] Man, age 64, T2DM and hypertension, > BMS, Spain

In Ecuador, participants emphasised the importance of having community networks or relationships that provide financial and emotional support:

…thank goodness I have a neighbor who gives me some work.

[ID26] Man, age 65, T2DM and hypertension, < BMS, Quito, Ecuador

…here in the parish there is a madre [religious person who lives in the community], she is diabetic too and whenever I call her we talk… She tells me do this ‘don't despair, don't get upset, you have to stay calm’.

[ID33] Woman, age 51, T2DM, Esmeraldas, Ecuador

Conceptual model determinants of the emotional state of people with diabetes and/or hypertension during the pandemic.

**Figure 1:**
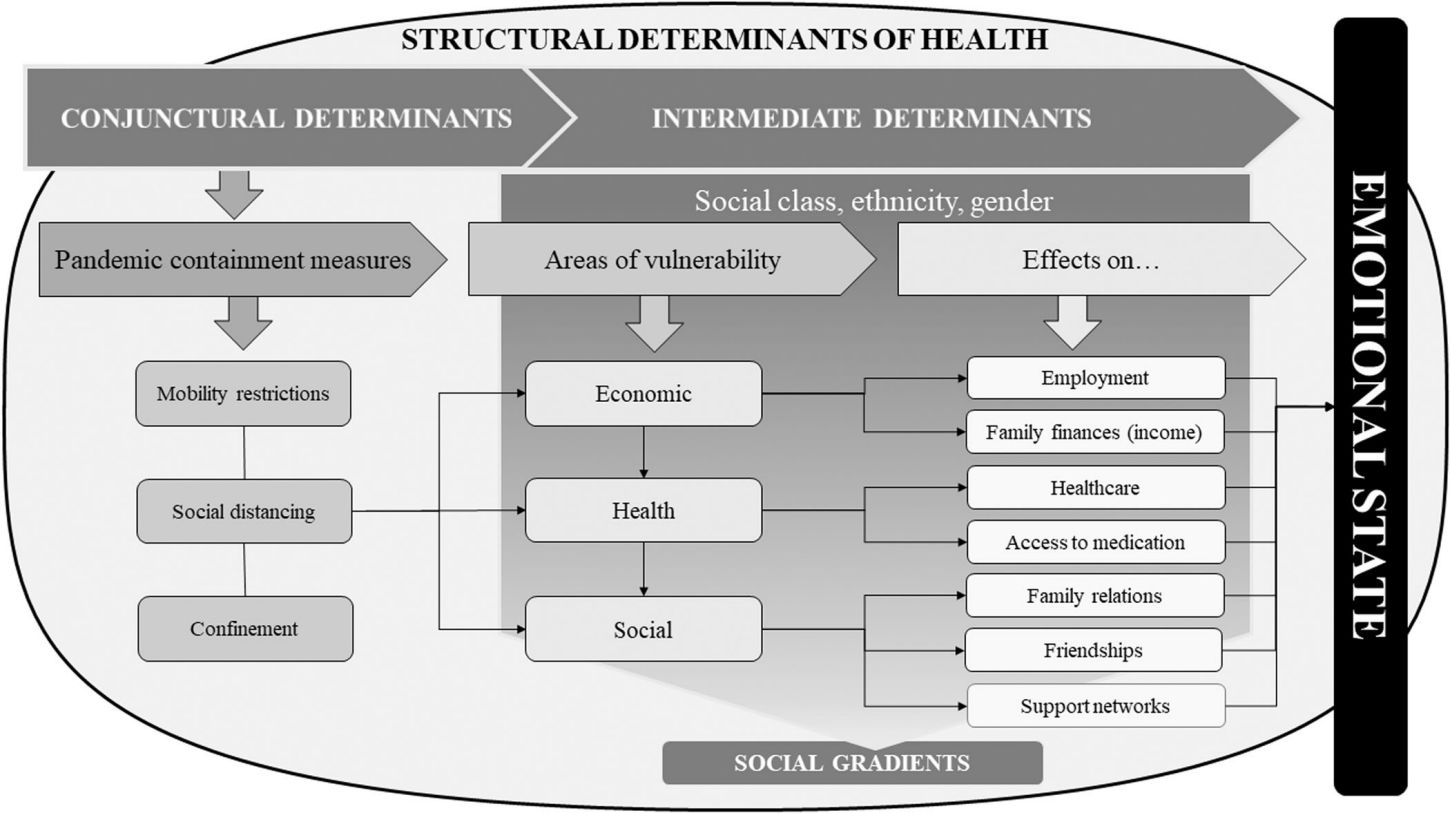
Conceptual model explaining the factors that determined the emotional state of people with diabetes and/or hypertension during the pandemic. *Source:* created by the authors.

Figure 1 proposes a conceptual framework explaining the determinants that modulate the emotional state of people with T2DM and/or hypertension during the pandemic in Ecuador and Spain. We show the process through which these determinants appeared to mediate the emotional state of individuals. Measures established by governments to prevent or control COVID‐19 infections, understood as conjunctural determinants, directly influenced intermediate (economic, health, social) determinants of health, which interacted with factors that drive inequalities (social class, sex, ethnicity) and had a differential impact on the emotional state of the people. All of this should be understood within the wider sociopolitical context of the country studied where characteristic such as welfare policies and other means of social protection (structural determinants) ultimately mould how these interactions influence their impact.

## 4 Discussion

The COVID‐19 pandemic and the associated containment measures, while necessary to control the spread of the virus, exacerbated vulnerabilities in key areas of life—economic, health and social—that disproportionately affected vulnerable groups such as older adults and those with chronic diseases like T2DM and hypertension [8, 10, 13]. This study explored the emotional state of people with T2DM and/or hypertension in the first months of the COVID‐19 pandemic in Ecuador and Spain. Our qualitative approach enabled us to explore the nuances of how economic and structural factors influenced participants' emotional states. While this methodology does not measure the extent of these influences, it captures the underlying dynamics and contextual factors shaping these experiences, providing a deeper understanding aligned with the principles of qualitative research.

Overall, participants experienced a marked deterioration in their emotional well‐being, consistent with other studies that have shown the compounding effects of chronic illness and the pandemic health crisis on psychological health [12, 15, 19]. Emotional responses ranged from boredom, apathy and/or nostalgia to fear, worry, uncertainty, anxiety, stress and depression, as the pandemic intensified participants' internalisation of their high‐risk status, further exacerbating feelings of vulnerability and fear of contagion [35, 36].

Our study found nuanced differences in emotional responses based on gender. While men expressed more concern about their own risk of infection and the economic repercussions of the pandemic, women were primarily concerned about infecting their loved ones, which is consistent with findings from similar studies in Spain and Serbia [11, 37]. This reflects the gendered nature of caregiving responsibilities, which disproportionately fall on women [3, 4, 5].

Our interviewees' responses varied according to geographical context and the rural or urban setting in Ecuador. These findings highlight how socioeconomic context shaped participants' emotional states, with those in Ecuador facing greater vulnerability due to economic challenges and healthcare barriers, while participants in Spain were more affected by isolation and confinement. Although we observed a deterioration in emotional state in both countries, participants in Ecuador expressed a greater feeling of vulnerability in the face of possible contagion, and greater concern, uncertainty and helplessness because of their financial situation, barriers to healthcare access and shortages of specific medications for T2DM and hypertension, particularly in rural settings. In Spain, interviewees' emotional state was characterised more by boredom, apathy and nostalgia caused by confinement and social distancing from family and friends.

The preventive measures prioritised by governments during the crisis had a range of consequences that revealed the fragility of existing economic and social policies [6] and were directly related to individuals' emotional states [7, 38]. These differences underscore the critical role of social and economic policies in mitigating the emotional impact of health crises, as seen in the contrasting experiences of participants in Ecuador and Spain. The loss, paralysis or reduction of work and salaries made it harder for people to access necessities and basic diet (particularly important for people with chronic illnesses); in settings without public and universal healthcare, people also had difficulty accessing medication and routine care [16, 17]. As a result, many felt socioeconomically unprotected [39]. These results could be attributable to the different economic development of the countries studied [40]. The pandemic significantly weakened and destabilised economies worldwide, particularly in developing countries like Ecuador, leading to an alarming socioeconomic situation. Between December 2019 and December 2020, Ecuador saw a more than 7‐percentage‐point increase in the poverty rate by income, rising from 25% to 32.4%.^1^ During this period, poverty affected an additional 1.3 million people in the country [41].

In line with previous studies, the vast majority of our participants perceived that the pandemic had seriously interrupted non‐communicable disease management services [15, 42]. In Ecuador, the situation generated by the health crisis affected the supply of T2DM and hypertension medications, which made people feel more vulnerable to stress and anxiety associated with the management of their disease [12]. This was not the case in Spain, where individuals diagnosed with T2DM and/or hypertension had continuous access to medication, which was also provided free of charge. As in other studies, the affected people themselves sometimes decided not to access healthcare services, possibly due to fear of infection [12, 13]. In other cases, the factor that limited healthcare access was lack of health coverage; in this context, access to medication and care depended on the economic situation of the individuals or families [6]. As some authors have pointed out, in the absence of effective public health interventions, socioeconomically vulnerable patients are less likely to adhere to treatment [43], so become acutely aware of their at‐risk status and more worried about becoming infected by COVID‐19 [10]. People with T2DM in our study felt more or less vulnerable in the face of possible COVID‐19 infection depending on their environment or country of residence. As in previous studies, some people felt that the health service offered no protection and that they had to take full charge of their illness [39]. This ultimately led to a feeling of helplessness in the face of a situation they could not control (socioeconomic restrictions and limited access to medical care, medication and/or healthy food).

Similarly to how the World Health Organization indicated that social, environmental and economic determinants influence and modulate people's health and lead to health inequities, it also stated that ‘many COVID‐19 containment measures, while beneficial for reducing infection risks, have immediate and potentially long‐term consequences for equity due to their adverse impact on key social determinants’ [44]. Thus, measures that we may consider temporary (implemented at a specific time and for a specific reason) have also modulated the impact of the pandemic on the emotional state of individuals with non‐communicable chronic diseases [45]. The interviewees with T2DM and/or hypertension also perceived a situation of social vulnerability because of the rupture caused by the pandemic in their family and social relationships, as well as the sudden interruption of their daily routines and their usual lifestyle [38]. Lockdowns and social distancing made some people feel lonely. Previous studies have found that loneliness increases vulnerability in at‐risk groups, who begin to neglect healthy behaviours, to the further detriment of their psychological and emotional well‐being [13]. According to other studies, social support is a direct predictor of quality of life in people with chronic diseases [6]. Many of our participants expressed having lost that social support because they had limited access to their family and friendship networks. In some cases, this perception was linked to loneliness and the feeling of being trapped in time (critical phase of the pandemic) and a limited space (small houses without a garden), as well as to the lack of financial and health protection. These factors lead to stress [10, 11, 12, 18], anxiety, apathy and/or nostalgia and a clear worsening of quality of life.

## 5 Limitations

This study has a series of limitations. Mobility restrictions and social distancing prevented in‐person interviews, leading us to conduct telephone interviews, which limited our ability to capture non‐verbal cues of participants' emotions. However, the interviewers did take notes on voice intonation and mood at certain points in the interview (e.g., during interview ID28, the interviewer noted ‘woman crying’). In addition, empirical evidence suggests that telephone interviews can offer comfort, privacy and anonymity, promoting sincerity and rich contributions [46].

Another limitation is related to the possible underrepresentation of specific profiles in Ecuador. We could not recruit people with diverse profiles in terms of education, profession and salary level since people with a low‐income level had limited access to the mobile network. Consequently, most rural participants had completed secondary education and had mid‐level jobs with a salary equal to or higher than the BMS. In Spain, we achieved a diverse profile most participants were retired and received a pension, so the sample was relatively homogeneous in terms of income. Additionally, participants were not recruited from rural areas in Spain, which face significant social, economic and healthcare system challenges, this could be explored in future research.

Furthermore, we lack information on the emotional state of interviewees before the pandemic, leaving us unable to assess any pre‐existing mental health issues, such as anxiety or depression, that may have worsened due to pandemic‐related circumstances. Similarly, the interview guide did not include direct questions about whether participants or their family members were affected by COVID‐19. This omission may have influenced the findings, as such experiences could have affected participants' emotional responses during the study period.

The two samples in this study (from Spain and Ecuador) represent different socioeconomic and political contexts, characterised by significant differences in social protection policies, including economic benefits and publicly funded healthcare. While this is not necessarily a limitation, it is a key aspect that readers should bear in mind when interpreting our results. One previous study by the International Diabetes Federation revealed that people in South and Central America face high unemployment, inadequate economic support from the government, and no universal health coverage, severely impacting chronic disease management [39, 47]. Tudor Hart proposed the inverse care law (1971), which clearly remains relevant today. Our results indicate a vicious circle: the absence of truly universal public healthcare forces financially disadvantaged people to seek private health plans, buy medications or pay for sporadic medical consultations. This situation exacerbates their socioeconomic conditions, further deteriorating their health and ultimately increases health inequality.

## 6 Conclusion

The deterioration in the quality of life among people with chronic non‐communicable diseases during the COVID‐19 pandemic cannot be separated from their financial, social and health status [6]. Different levels of welfare (social and health policies) translated into different emotional states in people with T2DM and/or hypertension. The effect of the health crisis on participants' emotional state varied depending on the economic, health and/or social dimension studied, all influenced by the conjunctural determinants arising from the pandemic.

This implies that, in health crisis situations, social policies must consider the needs of the most vulnerable risk groups, such as those with chronic diseases. When designing prevention and containment measures, it is crucial to consider the influence and possible effects on the emotional state of at‐risk people, aiming to preserve both their psychological and physical health. This approach would help to correct some of the health inequalities caused by the (unfair and unwanted) social and economic inequalities that disadvantage the most vulnerable groups.

## Author Contributions

**María José Sanchís‐Ramón:** investigation, conceptualisation, methodology, formal analysis, data curation, visualisation, writing–review and editing, writing–original draft, supervision, resources, software. **Elisa Chilet‐Rosell:** conceptualisation, formal analysis, visualization, writing–original draft. **Andrés Peralta:** visualization, writing–original draft. **Marta Puig‐García:** formal analysis, writing–original draft, data curation, visualization, investigation. **María Fernanda Rivadeneira:** writing–original draft, visualization. **Cintia Caicedo:** writing–original draft, visualization. **Ikram Benazizi‐Dahbi:** writing–original draft, visualization, conceptualisation, investigation, formal analysis, data curation. **Blanca Lumbreras:** writing–original draft, visualization. **Montse Nicols:** writing–original draft, visualization. **Ana Cebrián:** writing–original draft, visualization. **Wifredo Ricart:** writing–original draft, visualization. **Lucy A. Parker:** investigation, writing–original draft, writing–review and editing, conceptualisation, funding acquisition, visualization, resources, project administration, data curation, validation, software.

## Ethics Statement

All participants provided oral informed consent. The study was approved by the Research Ethics Committee of Sant Joan d'Alacant Hospital, the Research Ethics Committee of the University Hospital of Girona Doctor Josep Trueta, and the Human Research Ethics Subcommittee of Ecuador Central University.

## Conflicts of Interest

The authors declare no conflicts of interest.

## Supporting information

Supporting information.

Supporting information.

## Data Availability

The data that support the findings of this study are available from the corresponding author upon reasonable request. All data were handled in accordance with the applicable Spanish and Ecuadorian regulations, and specifically the Additional Provision 17 of Spanish Organic Law 3/2018, of 5 December, on the Protection of Personal Data and the Guarantee of Digital Rights.

## Endnotes

1 INEC. Boletín técnico N° 02‐202‐ENEMDU diciembre 2020 wwwecuadorencifras. gob. es
