# Supplementary material for: Social Determinants as Mediators of the Emotional State of People With Type 2 Diabetes and/or Hypertension During the COVID‐19 Pandemic in Ecuador and Spain
Source: Health Expect. 2024 Dec 11;27(6):e70123. doi: 10.1111/hex.70123 (PMC11632625; doi:10.1111/hex.70123)
Supplement: Supplementary file 1 — Supporting information. [file HEX-27-e70123-s002.docx]

Supplementary file 1: Interview guide

**1.**   **Socio-demographic/ socio-economic information:**

To begin with, I would like you to tell me a little bit about yourself. Prompts:

- How old are you?
- What is your marital status?
- What is your level of education, or what level did you achieve at school?
- What is your profession? What is your or your household's typical monthly income?
- With whom do you live, and do you have dependants?

**2.**      **Previous health experience:** What was your experience with your diabetes/hypertension like before the pandemic? Prompts:

- How have you managed the disease since you were diagnosed, who do you consult when you need help or advice (doctor, family, friends...)?
- What was your access to health services like, how often did you use them?
- Before the pandemic, did you ever have to stop managing your hypertension/diabetes or taking medication? If so, for what reason?
- How was your diabetes/hypertension controlled before the pandemic, how long have you had the disease, who do you usually consult when you need help or advice (doctor, family, friends, etc.)?
- Before the pandemic, could you routinely afford to buy the medicines or other products necessary for your health care?
- Where do you check your diabetes/hypertension, how easy was it for you to go there, did you go there often?

**3.**      **Management of illness during confinement:** I would like you to tell me about how confinement has affected living with your illness. Prompts:

- Do you feel that your health has improved, stayed the same or got worse, for what reason?
- Did you have someone to turn to in case of doubt or when you felt unwell, did you have any complications, how did you cope with them?
- Did you ever fail to attend health services even when you thought it was necessary?
- Did you stop taking any medication, for what reason?
- Has your diet changed during confinement, why?
- How has your physical activity changed as a result of the confinement, and why?

**4.**      **Personal experience:** How did you feel during the confinement, why did you feel this way? Prompts:

- Were you afraid of your illness or of going to the health centre, did you have someone to talk to about your concerns?
- How has the situation affected you and your family, how has the confinement affected your relationship with your family?
- How has your financial situation been affected?
- Has your workload in the household increased, how?
- Have you received any financial and social support in confinement?
