# Supplementary material for: Social Determinants as Mediators of the Emotional State of People With Type 2 Diabetes and/or Hypertension During the COVID‐19 Pandemic in Ecuador and Spain
Source: Health Expect. 2024 Dec 11;27(6):e70123. doi: 10.1111/hex.70123 (PMC11632625; doi:10.1111/hex.70123)
Supplement: Supplementary file 2 — Supporting information. [file HEX-27-e70123-s001.docx]

Supplementary file 2: Table 1, Sociodemographic characteristics of the participants

Table 1, Sociodemographic characteristics of the participants

| **ID** | **Age** | **Sex** | **Education** | **Ocupation** | **Monthly Salary in €** | **Marital Status** | **Cohabitants in the home** | **Disease duration in years** | **Location** | |
| --- | --- | --- | --- | --- | --- | --- | --- | --- | --- | --- |
| 01 | 62 | Female | Primary | Housewife | 1, 400.00 | Married | 4 | 5 | San Juan | Spain |
| 02 | 71 | Male | Superior | Technical Engineer | 2,000.00 | Married | 4 | 21 | Cartagena | Spain |
| 03 | 62 | Male | Secondary | Commercial | 1,300.00 | Widowed | 1 | 6 | Alzira | Spain |
| 04 | 75 | Female | Primary | Not reported | 800.00 | Widowed | 1 | 25 | Alzira | Spain |
| 05 | 62 | Female | Primary | Home assistant | 750.00 | Single | 4 | 10 | Cartagena | Spain |
| 06 | 80 | Male | Superior | Military doctor | 2,683.34 | Married | 2 | 40 | Cartagena | Spain |
| 07 | 55 | Male | No formal education | Farmer | No income | Single | 1 | 10 | Alzira | Spain |
| 08 | 64 | Female | Primary | Housewife | 1,400.00 | Married | 2 | 34 | Cartagena | Spain |
| 09 | 57 | Female | Primary | Pool assistant | 1,300.00 | Married | 2 | 6 | Alzira | Spain |
| 10 | 84 | Female | Secondary | Housewife | + 2,000.00 | Married | 2 | 4 | Girona | Spain |
| 11 | 57 | Male | Primary | Hospital attendant | + 2,000.00 | Married | 2 | Lifetime | Girona | Spain |
| 12 | 68 | Male | Primary | Mechanic | 646.00 | Single | 1 | 14 | Girona | Spain |
| 13 | 73 | Female | Primary | Housewife | + 1,000.00 | Married | 2 | 43 | Cartagena | Spain |
| 14 | 67 | Female | Primary | Administrative assistant | + 2,000.00 | Married | 3 | Not reported | Cartagena | Spain |
| 15 | 69 | Male | Superior | Surgeon | Not reported | Widowed | 1 | Not reported | Girona | Spain |
| 16 | 64 | Male | Superior | Accountant | 1,400.00 | Divorced | 1 | 15 | Cartagena | Spain |
| 17 | 52 | Female | Primary | Supermarket cashier | 1,400.00 | Widowed | 3 | 1 | Alzira | Spain |
| 18 | 61 | Male | Secondary | Farmer | 262.65 | Married | 5 | 5 | Esmeraldas | Ecuador |
| 19 | 67 | Male | Primary | Farmer | 122.57 | Divorced | 3 | Not reported | Quito | Ecuador |
| 20 | 50 | Male | Primary | Construction worker | 218.87 | Married | 4 | 12 | Quito | Ecuador |
| 21 | 63 | Female | Primary | Housewife | 175.10 | Married | 3 | Not reported | Quito | Ecuador |
| 22 | 43 | Female | Superior | Teacher | 617.22 | Married | 12 | 6 | Esmeraldas | Ecuador |
| 23 | 53 | Male | Secondary | Taxi driver | No income | Married | 5 | 21 | Quito | Ecuador |
| 24 | 50 | Female | Superior | Street vendor | 87.55 | Married | 4 | 15 | Quito | Ecuador |
| 25 | 48 | Female | Secondary | Book consultancy | Not reported | Not reported | 3 | 2 | Quito | Ecuador |
| 26 | 65 | Male | Secondary | Printing service | 262.65 | Married | 6 | 21 | Quito | Ecuador |
| 27 | 58 | Male | Secondary | Laboratory Assistant | 525.30 | Single | 8 | 18 | Esmeraldas | Ecuador |
| 28 | 55 | Female | Secondary | Street vendor | No income | Married | 2 | 15 | Quito | Ecuador |
| 29 | 52 | Female | Primary | Unemployed | No income | Married | 7 | 4 | Quito | Ecuador |
| 30 | 53 | Female | Secondary | Cleaner | 218.87 | Single | 1 | Not reported | Quito | Ecuador |
| 31 | 56 | Male | Secondary | Nursing Assistant | 507.79 | Non- marital union | 5 | 29 | Esmeraldas | Ecuador |
| 32 | 73 | Female | Primary | Housewife | 87.55 | Non- marital union | 3 | 15 | Esmeraldas | Ecuador |
| 33 | 51 | Female | Superior | Teacher | 367.71 | Married | 8 | 18 | Esmeraldas | Ecuador |
| 34 | 63 | Male | Superior | Unemployed | Not reported | Divorced | 1 | 20 | Quito | Ecuador |
| 35 | 70 | Male | Secondary | Commercial | 700.40 | Married | 3 | 10 | Quito | Ecuador |
| 36 | 59 | Male | Primary | Construction worker | Not reported | Married | 5 | 17 | Quito | Ecuador |
